# Clinical Advances in Breast Cancer: A Comprehensive Overview of Full‐Course Disease Management

**DOI:** 10.1002/cai2.70076

**Published:** 2026-08-19

**Authors:** Xuenan Peng, Yujing Tan, Fei Ma

**Affiliations:** ^1^ Department of Medical Oncology, National Cancer Center, National Clinical Research Center for Cancer, Cancer Hospital Chinese Academy of Medical Sciences and Peking Union Medical College Beijing China

**Keywords:** breast cancer, endocrine therapy, immunotherapy, targeted therapy, treatment advancement

## Abstract

The landscape of breast cancer (BC) treatment has reached a pivotal turning point in recent years, marked by a succession of breakthrough findings from landmark international clinical trials. These advances have fundamentally reshaped treatment paradigms across distinct molecular subtypes. In the field of hormone receptor‐positive (HR‐positive)/human epidermal growth factor receptor 2‐negative (HER2‐negative) BC, the introduction of novel targeted agents has continuously optimized therapeutic strategies. Meanwhile, the management of endocrine therapy resistance has evolved into an innovative framework characterized by multitarget therapeutic combination approaches. Concurrently, the extensive impact of the DESTINY‐Breast series has constructed a stratified, precision medicine system covering the entire disease course of HER2‐positive BC. In triple‐negative breast cancer, the ASCENT‐03 and ASCENT‐04 trials have successfully positioned antibody‐drug conjugates as a cornerstone of first‐line treatment for advanced disease. This article focuses on the latest advances in BC, systematically reviewing recent clinical evidence and practical experience in medical oncology to provide a robust reference for clinical diagnosis and treatment.

## Graphical abstract

This review provides a comprehensive overview of clinical advances in breast cancer, highlighting subtype‐specific therapeutic paradigms. It underscores the full‐cycle integration of innovative therapies, as well as the growing role of biomarker‐guided strategies in advancing precision medicine. (The graphical abstract image was Created in BioRender. Yaru, N. (2026) https://BioRender.com/pxekv8a)

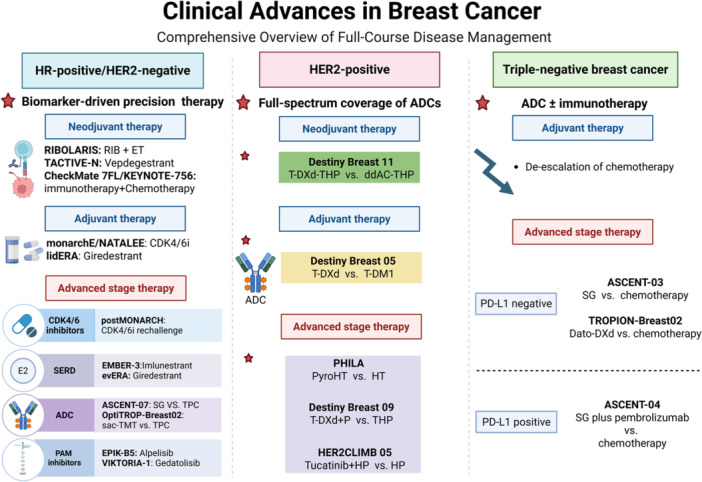

**Abbreviations**
ABC: advanced breast cancer
ADC: antibody‐drug conjugate
AE: adverse event
AI: aromatase inhibitor
AKT, also known as PKB: protein kinase B
ASCO: American Society of Clinical Oncology
BCFI: breast cancer‐free interval
BICR: blinded independent central review
CDK4/6i: cyclin‐dependent kinase 4/6 inhibitor
CI: confidence interval
DFS: disease‐free survival
DRFS: distant recurrence‐free survival
EBC: early breast cancer
EFS: event‐free survival
ER: estrogen receptor
ESMO: European Society for Medical Oncology Congress
ET: endocrine therapy
HER2: human epidermal growth factor receptor 2
HR: hazard ratio
HR: hormone receptor
iDFS: invasive disease‐free survival
ILD: interstitial lung disease
mTOR: mammalian target of rapamycin
NAT: neoadjuvant therapy
NET: neoadjuvant endocrine therapy
OFS: ovarian function suppression
ORR: objective response rate
OS: overall survival
pCR: pathological complete response
PFS: progression‐free survival
PI3K: phosphoinositide 3‐kinase
QoL: quality of life
RIB: ribociclib
sac‐TMT: sacituzumab tirumotecan
SERD: selective estrogen receptor degrader
SG: sacituzumab govitecan
TAM: tamoxifen
TKI: tyrosine kinase inhibitor
TNBC: triple‐negative breast cancer
TRAE: treatment‐related adverse event
TROP2: trophoblast cell surface antigen 2
T‐DM1: trastuzumab emtansine
T‐DXd: trastuzumab deruxtecan

## 1 Introduction

Breast cancer (BC) ranks first in incidence among malignancies in women worldwide and remains one of the cancer types with the greatest global disease burden [1]. Since the 2011 St. Gallen International Breast Cancer Conference established subtype classification criteria based on hormone receptor (HR) and human epidermal growth factor receptor 2 (HER2) status, the molecular subtype‐based diagnostic and therapeutic framework for HR‐positive/HER2‐negative, HER2‐positive, and triple‐negative breast cancer (TNBC) has been continuously refined. The establishment and evolution of this framework have substantially advanced precision medicine across the entire continuum of BC treatment and driven an ongoing paradigm shift in clinical practice.

HR‐positive/HER2‐negative BC accounts for 65%–70% of all BC cases. Endocrine therapy (ET) remains the cornerstone of treatment for both early‐stage BC and advanced breast cancer (ABC) [2]. In recent years, the introduction of novel targeted agents, including various inhibitors of the phosphoinositide 3‐kinase (PI3K)/protein kinase B (AKT)/mammalian target of rapamycin (mTOR) signaling pathway (PAM pathway), and antibody‐drug conjugates (ADCs), has continuously optimized treatment strategies. These advances have largely expanded therapeutic options for ET‐resistant disease [3, 4, 5]. In HER2‐positive BC, novel therapeutic strategies such as anti‐HER2 ADCs and HER2 tyrosine kinase inhibitors (TKIs) have led to marked improvements in survival outcomes [6, 7]. In TNBC, a highly aggressive BC subtype, while cytotoxic chemotherapy has traditionally dominated the treatment landscape, its efficacy is often constrained by limited long‐term benefit. In recent years, the broad integration of immunotherapy and ADCs has paved the way for transformative therapeutic strategies, offering promising new horizons for TNBC management [8].

In recent years, breakthroughs from several landmark international clinical trials have emerged, promoting a reconstruction of treatment paradigms across different molecular subtypes. This review focuses on pivotal advances in medical oncology and aims to systematically summarize the latest clinical evidence and practical insights to inform therapeutic decision‐making.

## 2 HR‐Positive/HER2‐Negative BC

The development of diverse novel therapeutic agents for patients with HR‐positive/HER2‐negative BC has reshaped the landscape of ET‐based treatment and post‐cyclin‐dependent kinase 4/6 inhibitor (CDK4/6i) therapy. Second‐generation oral selective estrogen receptor degraders (SERDs), inhibitors targeting the PAM pathway, ADCs, and proteolysis‐targeting chimera (PROTAC)‐based therapies have achieved breakthroughs in both early‐stage BC and ABC. The management of HR‐positive/HER2‐negative BC is progressively evolving toward a new paradigm of individualized, whole‐course care.

### 2.1 Progress in Neoadjuvant Therapy (NAT) for HR‐Positive/HER2‐Negative BC

For patients with HR‐positive/HER2‐negative localized BC (stages II and III), neoadjuvant chemotherapy remains the standard treatment option. However, the pathological complete response (pCR) rate to neoadjuvant chemotherapy is generally lower than that in other subtypes, while treatment is frequently accompanied by systemic toxicity [9]. An increasing number of studies have indicated that neoadjuvant endocrine therapy (NET) not only achieves clinical response rates comparable to those of chemotherapy but also demonstrates superior safety and quality‐of‐life (QoL) profiles. In 2025, the neoadjuvant landscape for high‐risk HR‐positive/HER2‐negative early breast cancer (EBC) advanced along two parallel tracks. In addition to further consolidating the role of NET in facilitating breast‐conserving surgery, significant breakthroughs in neoadjuvant immunotherapy have simultaneously expanded the therapeutic landscape, collectively providing more personalized and effective strategies for high‐risk subgroups. A comprehensive summary of the relevant studies is presented in Table 1.

**Table 1:** Summary of landmark clinical trials of breast cancer.

Molecular subtype	Setting	Trial	Therapeutic regimen	Major clinical outcomes	References
HR‐positive/HER2‐negative	Neoadjuvant	RIBOLARIS	Ribociclib + letrozole	52.6% of clinically high‐risk patients achieved a low recurrence risk score presurgery	[10]
		TACTIVE‐N	Vepdegestrant (oral PROTAC) versus anastrozole	Vepdegestrant decreased Ki‐67 by 84.5% after treatment completion, with a radiographic response rate of 41% and a breast‐conserving surgery rate of 70%	[11]
		CheckMate 7FL	Nivolumab + chemotherapy versus placebo + chemotherapy	Significantly higher pCR with nivolumab (24.5% vs. 13.8%), especially in PD‐L1 positive tumors (44.3% vs. 20.2%)	[12]
		KEYNOTE‐756	Pembrolizumab + chemotherapy versus placebo + chemotherapy	Pembrolizumab arm demonstrated a significantly superior pCR rate of 24.3% versus 15.6% (*p* = 0.00005)	[13]
	Adjuvant	SOFT/TEXT	OFS + exemestane versus OFS + tamoxifen (TAM) versus TAM alone	Exemestane + OFS showed sustained DFS benefit over TAM + OFS and TAM alone (15‐year DFS: 73.5% vs. 70.5% vs. 67.0%; HR = 0.82)	[14]
		monarchE	Abemaciclib (2 years) + ET versus ET alone	Abemaciclib reduced death risk by 15.8% with a 7‐year OS of 86.6% versus 85.0%	[15]
		NATALEE	Ribociclib (3 years) + NSAI versus NSAI alone	5‐Year data showed a 28.4% reduction in iDFS event risk (HR = 0.716), with sustained benefits in N0/N(+) disease	[16]
		lidERA	Giredestrant (oral SERD) versus standard ET	3‐Year iDFS rate was 92.4% versus 89.6% (HR = 0.70); giredestrant reduced distant metastasis risk by 31%	[17]
	Advanced	RIGHT Choice	RIB + ET versus combination chemotherapy	Achieved a 9‐month PFS benefit overall; extended median PFS by 5.6 months in liver metastases	[18]
		CULMINATE‐2	Culmerciclib (CDK2/4/6i) + fulvestrant versus fulvestrant	Significantly prolonged PFS in ET‐resistant ABC (NR vs. 20.2 months; HR = 0.40) with low grade 3/4 neutropenia	[19]
		SERENA‐6	Switch to camizestrant + CDK4/6i versus continue AI + CDK4/6i	Pre‐progression switch upon ESR1 mutation extended median PFS from 9.2 to 16.0 months (HR = 0.44)	[20]
		INAVO120	Inavolisib + palbociclib + fulvestrant versus palbociclib + fulvestrant	Extended median OS by 7 months (34.0 vs. 27.0 months; HR = 0.67) in *PIK3CA*‐mutated, ET‐resistant ABC	[4]
		postMONARCH	CDK4/6i rechallenge: abemaciclib plus fulvestrant	The median PFS was 6 months	[21]
		EMBER‐3	Imlunestrant (oral SERD) ± abemaciclib versus standard ET	Single‐agent reduced progression risk by 38% in *ESR1*; combination with abemaciclib reduced risk by 52% in dual *ESR1/PI3K* mutants	[22, 23]
		evERA	Giredestrant + everolimus versus standard ET + everolimus	Prolonged median PFS (8.77 vs. 5.49 months; HR = 0.56), with a 62% risk reduction in *ESR1*‐mutant disease	[24]
		EPIK‐B5	Alpelisib + fulvestrant versus placebo + fulvestrant	Improved median PFS (7.4 vs. 2.8 months; HR = 0.52) and prolonged median OS (29.5 vs. 23.8 months)	[25]
		VIKTORIA‐1	Gedatolisib + fulvestrant ± palbociclib versus fulvestrant	*PIK3CA* wild‐type: triplet achieved median PFS of 9.3 months (HR = 0.24); doublet achieved 7.4 months (HR = 0.33)	[26]
		Nab‐sirolimus	Nab‐sirolimus + ET	The median PFS was 5.7 months regardless of specific PAM pathway mutation status	[27]
		ASCENT‐07	Sacituzumab govitecan (SG) versus chemotherapy	Investigator‐assessed outcomes revealed a significant PFS benefit favoring SG over conventional chemotherapy (8.4 vs. 6.4 months; HR = 0.78; *p* = 0.008)	[28]
		OptiTROP‐Breast 02	sac‐TMT (TROP‐2‐ADC) versus chemotherapy	In heavily pretreated ABC, sac‐TMT doubled median BICR‐PFS (8.3 vs. 4.1 months; HR = 0.35)	[29]
HER2‐positive	Neoadjuvant	neoCARHP	THP versus TCbHP	THP matched TCbHP pCR rates (64.1% vs. 65.9%), significantly reducing grade 3/4 AEs	[30]
		DESTINY‐Breast 11	THP‐T‐DXd versus ddAC‐THP	T‐DXd‐THP achieved a superior pCR rate of 67.3% versus 56.3% (*p* = 0.003) with a better safety profile	[31]
	Adjuvant	DESTINY‐Breast 05	T‐DXd versus T‐DM1 (residual invasive disease post‐NAT)	T‐DXd reduced iDFS event or death risk by 53% (HR = 0.47); 3‐year iDFS rate was 92.4% versus 83.7%	[32]
	Advanced	PHILA	Pyrotinib + trastuzumab + docetaxel (PyroHT) versus HT	At 46.5 months follow‐up, the PyroHT arm maintained a significant OS advantage (HR = 0.74; *p* = 0.0188)	[33]
		DESTINY‐Breast 09	T‐DXd + pertuzumab versus standard THP	PFS: 40.7 months for T‐DXd + P versus 26.9 months for THP (HR = 0.56; *p* < 0.00001)	[34, 35, 36]
		HER2CLIMB‐05	Tucatinib + HP versus placebo + HP (maintenance)	Adding tucatinib to first‐line HP maintenance significantly prolonged PFS (HR = 0.641; *p* < 0.0001)	[37]
		HORIZON‐Breast 01	SHR‐A1811 versus pyrotinib + capecitabine	SHR‐A1811 achieved a massive median PFS of 30.6 months versus 8.3 months in the control (HR = 0.22)	[38]
		A166	Trastuzumab botidotin (A166) versus T‐DM1	A166 extended mPFS from 4.4 to 11.1 months (HR = 0.39), with reduced hematologic/hepatic toxicities	[39]
TNBC	Neoadjuvant	HELEN‐001	Docetaxel + cisplatin (TP) versus TAC	TP achieved a higher pCR rate (51.9% vs. 35.8%)	[40]
		PLANeT	Low‐dose pembrolizumab (50 mg) + NACT versus NACT	Low‐dose arm reached a 53.8% pCR rate versus 40.5% in control, with slightly lower grade ≥ 3 AEs	[41]
	Adjuvant	NRG‐BR003	AC‐wP + carboplatin versus AC‐wP alone	Carboplatin intensification failed to improve 5‐year iDFS (82.7% vs. 77.8%; *p* = 0.12) with higher toxicity	[42]
		RJBC‐1501	EC‐T + carboplatin (EC‐TCb) versus EC‐T	Carboplatin significantly improved 3‐year DFS (93.1% vs. 89.8%), dDFS, and overall OS	[43]
		CITRINE	ddEC‐PCb (carboplatin intensified) versus control	Carboplatin improved 3‐year DFS (92.3% vs. 85.8%) and OS (98.0% vs. 94.0%), concentrated in the first year	[44]
	Advanced	ASCENT‐03	SG versus chemotherapy	SG significantly prolonged survival (mPFS 9.7 vs. 6.9 months, HR = 0.62)	[45]
		TROPION‐Breast 02	Datopotamab deruxtecan (Dato‐DXd) versus chemotherapy	Dato‐DXd extended median PFS by 5.2 months (HR = 0.57) and improved median OS by 5 months	[46]
		OptiTROP‐Breast 05	Sacituzumab tirumotecan	First‐line sacituzumab tirumotecan deployment achieved an impressive median PFS of 13.4 months with manageable safety	[47]
		ASCENT‐04	SG + pembrolizumab versus chemotherapy + pembrolizumab	In PD‐L1 positive ABC, combination extended median PFS to 11.2 months versus 7.8 months (HR = 0.65) with lower discontinuation	[48]
		BEGONIA	Dato‐DXd + durvalumab	Achieved a 79.0% ORR overall and 14.0 months median PFS, showing strong survival benefits regardless of PD‐L1 status	[49]

Abbreviations: ABC, advanced breast cancer; AC‐wP, adriamycin plus cyclophosphamide followed by weekly paclitaxel; ADC, antibody‐drug conjugate; AEs, adverse events; AI, aromatase inhibitor; BICR, blinded independent central review; CDK2/4/6i, cyclin‐dependent kinase 2/4/6 inhibitor; CDK4/6i, cyclin‐dependent kinase 4/6 inhibitor; ddAC‐THP, dose‐dense adriamycin plus cyclophosphamide followed by taxane, trastuzumab, and pertuzumab; dDFS, distant disease‐free survival; DFS, disease‐free survival; EC‐T, epirubicin plus cyclophosphamide followed by taxanes; ET, endocrine therapy; HER2, human epidermal growth factor receptor 2; HR, Hazard Ratio; HR, hormone receptor; iDFS, invasive disease‐free survival; NACT, neoadjuvant chemotherapy; NR, not reached; NSAI, nonsteroidal AI; OFS, ovarian function suppression; ORR, objective response rate; OS, overall survival; pCR, pathological complete response; PFS, progression‐free survival; PROTAC, proteolysis‐targeting chimera; PyroHT, pyrotinib, trastuzumab, and docetaxel; sac‐TMT, sacituzumab tirocitinib; SERD, selective estrogen receptor degrader; TAC, docetaxel, adriamycin, and cyclophosphamide; TCbHP, docetaxel, carboplatin, trastuzumab, and pertuzumab; T‐DM1, trastuzumab emtansine; T‐DXd, trastuzumab deruxtecan; THP, taxane, trastuzumab, and pertuzumab; TNBC, triple‐negative breast cancer; TP, Docetaxel and Cisplatin; TROP‐2, trophoblast cell surface antigen‐2.

At the 2025 European Society for Medical Oncology Congress (ESMO), the RIBOLARIS study reported its initial results. This study evaluates recurrence risk in patients with high‐risk HR‐positive/HER2‐negative EBC treated with neoadjuvant ribociclib (RIB) plus letrozole for 24 weeks [10]. The study plans to enroll 1100 HR‐positive/HER2‐negative EBC patients at high risk of recurrence, characterized by clinicopathological features such as clinical stage II disease, histological grade 2/3, Ki‐67 ≥ 20% or a high‐risk genomic test result. On the basis of findings from the NeoPAL and CORALLEEN trials [50, 51], it was prespecified that 40% of patients should achieve a low recurrence risk score before surgery. The first interim analysis (*N* = 686) showed that 52.6% of patients were classified as low risk and 47.4% as high risk. These results indicate that patients with high‐risk HR‐positive/HER2‐negative EBC may achieve low‐risk status after 24 weeks of NET with RIB plus letrozole.

Novel PROTAC agents in the neoadjuvant setting have also demonstrated promising efficacy. Vepdegestrant is an oral PROTAC estrogen receptor (ER) degrader that directly exploits the ubiquitin‐proteasome system to efficiently degrade ER [52]. The TACTIVE‐N study evaluated vepdegestrant or anastrozole as NAT in postmenopausal women with ER‐positive/HER2‐negative EBC [11]. After 2 weeks of vepdegestrant treatment, the Ki‐67 index was reduced by 71.4%. Upon completion of NAT, the Ki‐67 index further decreased by 84.5%, with a radiographic response rate of 41% and a breast‐conserving surgery rate of 70%. In the anastrozole arm, Ki‐67 expression decreased by 72.9% at week 2 and by 82.8% at treatment completion, with a radiographic response rate of 42% and a breast‐conserving surgery rate of 54%. These findings suggest that vepdegestrant demonstrates promising clinical efficacy as NAT in treatment‐naïve postmenopausal patients with ER‐positive/HER2‐negative EBC.

Furthermore, immunotherapy has emerged as a promising strategy in the neoadjuvant management of high‐risk HR‐positive/HER2‐negative EBC. Two landmark phase III trials, CheckMate‐7FL [12] and KEYNOTE‐756 [13], evaluated the incorporation of immune checkpoint inhibitors (nivolumab and pembrolizumab, respectively) into standard anthracycline/taxane‐based neoadjuvant chemotherapy for grade 3 or high‐risk invasive disease. Importantly, both studies consistently demonstrated that the addition of immunotherapy significantly increased pCR rates to approximately 24%, nearly doubling the 14%–15% achieved by chemotherapy alone (CheckMate‐7FL, 24.5% vs. 13.8%, *p* = 0.0021; KEYNOTE‐756, 24.3% vs. 15.6%, *p* = 0.00005). Notably, CheckMate‐7FL further showed that this clinical benefit was most pronounced in PD‐L1‐positive tumors (44.3% vs. 20.2%) and those with elevated stromal tumor‐infiltrating lymphocytes. Collectively, these findings provide compelling high‐level evidence that highlights the critical role of immunotherapy even in traditionally luminal disease, thereby establishing a promising new therapeutic paradigm for high‐risk HR‐positive/HER2‐negative EBC.

### 2.2 Progress in Adjuvant Therapy for HR‐Positive/HER2‐Negative BC

The adjuvant therapeutic landscape for HR‐positive/HER2‐negative EBC has formally evolved from sequential endocrine monotherapy to a highly sophisticated paradigm characterized by risk‐stratified treatment escalation and holistic, patient‐centered survival management.

Mature long‐term follow‐up data from classic endocrine strategies remain the cornerstone of adjuvant care. The landmark 15‐year updates of the SOFT and TEXT trials have definitively solidified the clinical value of combining ovarian function suppression (OFS) with an aromatase inhibitor (AI) over tamoxifen (TAM) alone. At the 2025 American Society of Clinical Oncology (ASCO) Annual Meeting, the 15‐year disease‐free survival (DFS) rates reported in the SOFT trial were 67.0% for the TAM‐alone group, 70.5% for the TAM + OFS group, and 73.5% for the exemestane + OFS group [14]. Compared with TAM alone, TAM + OFS (Hazard Ratio [HR] = 0.85; 95% confidence interval [CI]: 0.72–1.00) and exemestane + OFS (HR = 0.73; 95% CI, 0.61–0.86) were associated with improved DFS. The 15‐year overall survival (OS) rates were 85.3%, 86.7%, and 86.9% for the TAM, TAM + OFS, and exemestane + OFS groups, respectively. The 15‐year BC‐free interval (BCFI) rates were 78.6% for exemestane + OFS, 75.7% for TAM + OFS, and 72.1% for TAM alone. The SOFT and TEXT trials represent landmark studies in adjuvant therapy for premenopausal patients with HR‐positive EBC, firmly establishing OFS combined with AI as a standard of care for high‐risk patients. These findings indicate that, among patients younger than 35 years, those with positive lymph nodes, or requiring adjuvant chemotherapy, the OFS + AI regimen significantly reduces the risk of recurrence compared with TAM alone.

The clinical application of novel targeted agents, particularly CDK4/6i, has substantially reshaped the therapeutic landscape for HR‐positive/HER2‐negative EBC, driving a paradigm shift from traditional endocrine monotherapy to combination targeted regimens and conferring meaningful survival benefits. The monarchE study showed that the addition of 2‐year adjuvant abemaciclib to ET significantly improved invasive DFS (iDFS) compared with ET alone in high‐risk, lymph node‐positive, HR‐positive/HER2‐negative patients, establishing this combination as a standard regimen for this population [53, 54]. In contrast to monarchE trial, the NATALEE study further expanded the target population for CDK4/6i by evaluating the efficacy and safety of a 3‐year adjuvant RIB plus AI regimen in a broader cohort of stages II and III patients (including stage IIA N0 disease with grade 3 histology, grade 2 with Ki‐67 ≥ 20%, or high‐risk genomic signatures) [55]. Updated follow‐up results from monarchE were presented at the 2025 ESMO Congress, with a median follow‐up of 76 months, and all patients had discontinued abemaciclib for at least 4 years. The results showed a 15.8% reduction in the risk of death with abemaciclib + ET versus ET alone (HR = 0.842; 95% CI, 0.722–0.981; *p* = 0.027). Landmark analysis at 7 years continued to show sustained benefits of abemaciclib + ET in both iDFS and distant recurrence‐free survival (DRFS), with a 26.6% reduction in the risk of iDFS events (HR = 0.734; 95% CI, 0.657–0.820; *p* < 0.0001) and a 25.4% reduction in the risk of DRFS events (HR = 0.746; 95% CI, 0.662–0.840; *p* < 0.0001) [15]. In the NATALEE trial, adjuvant RIB plus nonsteroidal AI significantly reduced the risk of an iDFS event by 28.4% (HR = 0.716; *p* < 0.0001), demonstrating sustained benefits in both high‐risk N0 (HR = 0.606) and node‐positive cohorts (HR = 0.737) [16]. On the basis of these findings, the 2025 editions of the Chinese Anti‐Cancer Association—Committee of Breast Cancer Society and the Chinese Society of Clinical Oncology—Breast Cancer guidelines have appropriately refined their risk stratification models, redefining the criteria for high, intermediate, and low recurrence risk patients.

Although the prevalence of germline *BRCA1/2* (g*BRCA1/2*) mutations is relatively low among patients with HR‐positive/HER2‐negative BC, carriers of these mutations with high‐risk EBC exhibit a significantly elevated risk of recurrence. Currently, for high‐risk patients harboring g*BRCA1/2* mutations, a 1‐year course of adjuvant intensification with olaparib is recommended. Updated analysis of the OlympiA trial showed a numerically sustained iDFS benefit with olaparib in the HR‐positive subgroup. Specifically, the 6‐year iDFS rate was 77.5% in the olaparib arm versus 67.7% with the placebo arm, representing a 9.8% absolute improvement and a 31.9% reduction in the risk of invasive disease recurrence or death (HR = 0.681; 95% CI, 0.437–1.051) [56]. However, for patients who simultaneously meet the criteria for both adjuvant CDK4/6i and poly (ADP‐ribose) polymerase inhibitor intensification, optimizing the treatment sequence and timing of CDK4/6i and poly (ADP‐ribose) polymerase inhibitor remains poorly defined. During the 2025 St. Gallen International Breast Cancer Consensus session, a clear majority of the expert panel favored a sequential administration strategy over concurrent combination, aiming to mitigate the potential risk of overlapping toxicities while effectively minimizing recurrence risk. Accordingly, defining the optimal management paradigm for this specific patient cohort remains an active and profound area of ongoing clinical investigation.

Furthermore, novel SERDs have shown therapeutic potential in the adjuvant setting. Results from the lidERA study were presented at the 2025 San Antonio Breast Cancer Symposium [17]. This trial compared giredestrant with standard ET (TAM or AI) as adjuvant treatment for patients with ER‐positive/HER2‐negative EBC. The 3‐year iDFS rate was 92.4% in the giredestrant group, superior to the 89.6% observed in the standard ET group (HR = 0.70; 95% CI, 0.57–0.87; *p* = 0.0014). Giredestrant also demonstrated superiority in DRFS, reducing the risk of metastatic disease by 31% (HR = 0.69; 95% CI, 0.54–0.89). The incidence of treatment‐related adverse events (TRAEs) (giredestrant vs. standard ET: 73.3% vs. 72.2%), ≥ grade 3 adverse events (AEs) (19.8% vs. 17.9%), and serious AEs (10.8% vs. 10.1%) was comparable between the two treatment arms.

The association between pregnancy and prognosis in young BC patients receiving adjuvant ET remains a subject of debate. The POSITIVE trial evaluated the safety and pregnancy outcomes following a temporary interruption of ET in young women (≤ 42 years) with stage I–III HR‐positive BC who desired pregnancy after suegery [57]. Participants temporarily suspended adjuvant ET for up to 2 years to attempt conception, delivery, and optional breastfeeding, before resuming ET to complete a total of 5–10 years of treatment. In the POSITIVE study, the 5‐year cumulative incidence of BCFI events was 12.3% and the 5‐year cumulative incidence of DRFS events was 6.2% for HR‐positive/HER2‐negative patients who interrupted ET for pregnancy. In a matched patient cohort from the SOFT/TEXT studies, the corresponding 5‐year cumulative incidence of BCFI events was 13.2%, and the 5‐year cumulative incidence of DRFS events was 8.3%. In the evaluable population of the POSITIVE study, 75.8% of participants had at least one pregnancy, with a live birth rate of 91% among those achieving pregnancy. The most common pregnancy complication was hypertension (2.5%). Low birth weight was observed in 8.6% of offspring, and birth defects occurred in 1.8%, with rates consistent with the general population. These results suggest that for young BC patients, a temporary interruption of ET for pregnancy did not increase the risk of BC events over a 5‐year follow‐up period.

### 2.3 Progress in Treatment for HR‐Positive/HER2‐Negative ABC

The therapeutic paradigm for HR‐positive/HER2‐negative ABC has evolved beyond static, empirical sequencing toward a highly precise, biomarker‐driven framework. Recent clinical insights have divided advanced management into two interconnected strategies: maximizing front‐line precision and addressing clonal evolution after CDK4/6i progression.

#### 2.3.1 First‐Line (1L) Treatment Strategy

In the first‐line setting, the clinical mandate has shifted from sequential cytotoxic chemotherapy to molecularly optimized targeted regimens, even in patients with a high tumor burden. The long‐term follow‐up of the RIGHT Choice trial definitively established first‐line RIB plus ET over chemotherapy, demonstrating an extensive progression‐free survival (PFS) benefit in patients with visceral (21.8 vs. 12.8 months, HR = 0.61; 95% CI, 0.43–0.87) and liver metastases (18.3 vs. 12.7 months, HR = 0.68; 95% CI, 0.42–1.11) while reducing exposure to chemotherapy‐associated systemic toxicities [18, 58]. In patients without liver metastases, RIB + ET versus chemotherapy led to a 43% reduction in the risk of disease progression or death, extending the median PFS by 9.8 months (25.2 vs. 15.4 months, HR = 0.57; 95% CI, 0.34–0.93). In terms of safety, chemotherapy was associated with a higher incidence of AEs than RIB + ET.

Concurrently, novel cell‐cycle inhibitors such as culmerciclib (TQB3616) are also being explored as potential front‐line treatment options by simultaneously targeting CDK2/4/6 to effectively delay endocrine resistance. The CULMINATE‐2 study aimed to evaluate the efficacy of first‐line culmerciclib plus fulvestrant versus fulvestrant monotherapy in patients with HR‐positive/HER2‐negative ABC [19]. The interim analysis showed that, in the intention‐to‐treat population, first‐line culmerciclib combined with fulvestrant significantly prolonged PFS in patients with ET‐resistant HR‐positive/HER2‐negative ABC (PFS by blinded independent central review [BICR]: not reached vs. 20.2 months, HR = 0.40; 95% CI, 0.27–0.59; *p* < 0.0001). Safety analysis indicated that culmerciclib plus fulvestrant presented a manageable safety profile, with a low discontinuation rate (3.5%) and a low incidence of ≥ grade 3 neutropenia (20.3%).

Crucially, rather than waiting for overt radiological progression, first‐line management is shifting toward proactive intervention. The liquid biopsy‐guided SERENA‐6 trial provided a landmark conceptual advance, demonstrating that the use of circulating tumor DNA to detect emerging *ESR1* mutations before disease progression enables a successful preemptive switch to camizestrant plus CDK4/6i [20]. Among patients with circulating tumor DNA‐detected *ESR1* mutations and no evidence of disease progression, median PFS was 16.0 months in those switched to camizestrant + CDK4/6i (*N* = 157) versus 9.2 months in those who continued AI + CDK4/6i (*N* = 158) (HR = 0.44; *p* < 0.00001). This PFS benefit was consistent across all CDK4/6i subgroups and clinically relevant subgroups. The median time from initiation of AI + CDK4/6i therapy to detection of *ESR1* mutations was 22 months.

In patients with *PIK3CA*‐mutated, ET‐resistant HR‐positive/HER2‐negative ABC, hyperactivation of the PI3K pathway represents a critical mechanism of resistance. The landmark phase III INAVO120 trial presented major paradigm‐shifting updates, validating that the addition of the PI3Kα‐selective inhibitor inavolisib to a palbociclib and fulvestrant backbone significantly prolonged median PFS by nearly 10 months (17.2 vs. 7.3 months; HR = 0.42; *p* < 0.0001) and increased the objective response rate (ORR) by an absolute 34.7% (62.7% vs. 28.0%). Inavolisib stands as the first PI3K pathway‐targeted agent to demonstrate a statistically significant and clinically meaningful OS benefit, extending median OS by 7 months (34.0 vs. 27.0 months; HR = 0.67; *p* = 0.019) [4]. These mature data support this biomarker‐directed triplet regimen as an essential front‐line standard for this genetically defined patient population.

#### 2.3.2 Post‐CDK4/6i Treatment Strategy

As the disease progresses after CDK4/6i therapy, clinical management requires precise biomarker‐guided treatment switching. Beyond traditional sequential chemotherapy, targeted interventions addressing abnormalities in the PAM pathway have emerged as core option. Concurrently, novel oral SERDs and proteolysis‐targeting chimera‐based agents, which leverage chemically induced proximity technology have expanded therapeutic options for endocrine‐resistant disease. Furthermore, ADCs, such as sacituzumab tirumotecan (sac‐TMT), provide profound survival benefits in the post‐first‐line setting. For specific patient subgroups, CDK4/6i rechallenge after prior CDK4/6i exposure or in novel combination regimens represents an important strategy for achieving long‐term disease control.

##### 2.3.2.1 CDK4/6i Rechallenge

CDK4/6i rechallenge remains a viable, albeit selective, strategy for patients who retain pathway dependence. In the postMONARCH trial, abemaciclib combined with fulvestrant achieved a median PFS of 6.0 months in patients with HR‐positive/HER2‐negative ABC who had previously progressed on CDK4/6i therapy [21]. However, only 8% of the study population had previously received abemaciclib. The phase II AGAIN trial evaluated the efficacy of abemaciclib rechallenge in combination with ET in patients with HR‐positive/HER2‐negative ABC following progression on abemaciclib‐based therapy [59]. A total of 65 patients were enrolled. In the overall population, the median PFS was 4.2 months (95% CI, 2.8–4.6), with an ORR of 8.7% (95% CI, 2.4–20.8) and a CBR of 23.9% (95% CI, 12.6–38.8). Among patients who progressed on abemaciclib plus AI/TAM and were subsequently switched to abemaciclib plus fulvestrant, the median PFS was 7.1 months (95% CI, 3.9–11.3). In contrast, among those who progressed on abemaciclib plus fulvestrant and were switched to abemaciclib plus AI/TAM, the median PFS was 2.8 months (95% CI, 1.9–4.2), and the median OS was 28.7 months (95% CI, 25.4–not reached).

##### 2.3.2.2 SERD

SERDs have demonstrated remarkable efficacy in disrupting ER signaling to overcome acquired *ESR1*‐mediated resistance after CDK4/6i failure [60]. In the phase III EMBER‐3 trial, the novel oral SERD imlunestrant significantly prolonged both PFS (5.5 vs. 3.8 months; HR = 0.62; *p* = 0.0007) and OS (34.5 vs. 23.1 months; HR = 0.60) compared with standard ET in the *ESR1*‐mutant population. Furthermore, the combination of imlunestrant and abemaciclib resulted in a 41% reduction in the risk of progression overall, with a particularly pronounced clinical benefit observed in patients harboring concurrent *ESR1* and *PI3K* pathway mutations (HR = 0.48) [22, 23]. Similarly, the phase III evERA trial confirmed the safety and efficacy of combining the oral SERD giredestrant with everolimus. This regimen established an effective dual‐pathway blockade and significantly prolonged median PFS compared with standard ET plus everolimus in the intention‐to‐treat population (8.77 vs. 5.49 months; HR = 0.56), with the benefit increasing to a 62% reduction in risk among the *ESR1*‐mutant subgroup (HR = 0.38). Collectively, these landmark studies solidify next‐generation oral SERDs—either as effective monotherapies or as components of rational targeted combinations‐as a vital therapeutic option for overcoming endocrine resistance [24].

##### 2.3.2.3 Inhibitors Targeted on the PAM Pathway

Hyperactivation of the *PI3K/AKT/mTOR* (PAM) signaling axis represents a hallmark mechanism of secondary resistance following progression on CDK4/6i therapy, providing a rationale for biomarker‐guided targeting of different nodes within the pathway. For patients with *PIK3CA*‐mutated disease, the phase III EPIK‐B5 trial established the role of the PI3Kα inhibitor alpelisib combined with fulvestrant, demonstrating statistically significant benefits in both median PFS (7.4 vs. 2.8 months; HR = 0.52; *p* < 0.0001) and OS (29.5 vs. 23.8 months; HR = 0.64) [25]. Further downstream in the signaling cascade, the phase III CCTG/BCT MA.40/FINER study established the *AKT* inhibitor ipatasertib plus fulvestrant as a superior regimen over endocrine monotherapy (PFS: 5.32 vs. 1.94 months; HR = 0.61), with clinical efficacy most pronounced in cohorts harboring alterations within the *AKT* pathway (HR = 0.47) [61]. To overcome individual genomic constraints and address *PIK3CA* wild‐type populations,the phase III VIKTORIA‐1 trial evaluated gedatolisib as a triplet with palbociclib and fulvestrant or as a doublet with fulvestrant in patients with *PIK3CA*‐wild‐type disease. Both regimens significantly improved PFS compared with fulvestrant alone, yielding substantial PFS benefits (triplet: 9.3 months, HR = 0.24; doublet: 7.4 months, HR = 0.33) with manageable toxicities [26]. The most common gedatolisib‐related AE was stomatitis (69.2% in the triplet therapy group; 56.9% in the doublet therapy group), predominantly grades 1 and 2 and manageable with interventions like mouthwash. Complementing these findings, preliminary data for the *mTOR* inhibitor nab‐sirolimus (albumin‐bound nanoparticle formulation of sirolimus, HB1901) plus ET demonstrated a median PFS of 5.7 months overall, indicating meaningful clinical utility in heavily pretreated cohorts regardless of specific PAM pathway mutation status [27]. Collectively, these studies illustrate the transition from empirical treatment sequencing after CDK4/6i progression toward biomarker‐guided targeting of specific nodes within the PAM pathway.

##### 2.3.2.4 ADCs

ADCs have also progressively demonstrated substantial clinical value in the post‐CDK4/6i salvage setting. The global phase III ASCENT‐07 trial evaluated the trophoblast cell surface antigen 2 (TROP2)‐directed ADC sacituzumab govitecan (SG) against treatment of physician's choice chemotherapy in a cohort where approximately 90% of patients had experienced disease progression following prior CDK4/6i therapy. Investigator‐assessed outcomes revealed a PFS benefit favoring SG over conventional chemotherapy (8.4 vs. 6.4 months; HR = 0.78; *p* = 0.008), complemented by an early favorable trend toward improved OS (HR = 0.72), despite a statistically nonsignificant difference under BICR assessment [28]. Consistent with these insights, the phase III OptiTROP‐Breast02 trial investigated the novel TROP‐2‐targeted ADC sac‐TMT versus physician's choice of chemotherapy in a similarly heavily pretreated population. Under BICR assessment, sac‐TMT significantly prolonged median PFS compared with standard chemotherapy (8.3 vs. 4.1 months; HR = 0.35; *p* < 0.0001) and achieving an absolute 17.4% improvement in ORR (41.5% vs. 24.1%), alongside a manageable safety profile predominantly characterized by expected hematologic toxicities [29]. These landmark trials support TROP2‐directed ADCs as important treatment options for patients with HR‐positive/HER2‐negative ABC following progression on CDK4/6i‐based therapy, offering clinically meaningful efficacy compared with conventional chemotherapy.

## 3 HER2‐Positive BC

The clinical treatment paradigm for HER2‐positive BC is undergoing a profound transition from “pathway antagonism” to “precision payload delivery.” Centered on pivotal trials such as DESTINY‐Breast 09, ADCs represented by trastuzumab deruxtecan (T‐DXd) are steadily establishing a core position in the first‐line treatment of advanced HER2‐positive BC. This transition challenges the THP (taxane, trastuzumab, and pertuzumab) regimen, which has remained the first‐line standard for over a decade. Furthermore, ADCs are gradually moving into the neoadjuvant and adjuvant settings, driving a comprehensive revolution in the therapeutic landscape of HER2‐positive BC.

### 3.1 Progress in NAT for HER2‐Positive BC

For early‐stage HER2‐positive BC, neoadjuvant regimens consisting of taxanes and carboplatin combined with trastuzumab and pertuzumab have demonstrated favorable therapeutic efficacy. However, the selection of traditional chemotherapy agents remains a focal point of research. Studies such as TRAIN‐2 [62], HELEN‐006 [63], and BCIRG‐007 [64] have explored strategies for “anthracycline‐free” and “platinum‐free” approaches while maintaining pCR rates and favorable long‐term outcomes, suggesting that chemotherapy de‐escalation has become the primary direction for future regimen selection.

At the 2025 ASCO Annual Meeting, the phase III neoCARHP study further investigated the feasibility of de‐escalated NAT by comparing the efficacy and safety of the THP regimen versus the TCbHP regimen in early‐stage HER2‐positive BC [30]. The study randomized 774 patients to receive either carboplatin‐containing (TCbHP) or carboplatin‐free therapy (THP) for six cycles. Regarding the primary endpoint, the pCR rates were 64.1% and 65.9% in the THP and TCbHP groups, respectively. Noninferiority testing showed an odds ratio with a 95% CI of 0.69–1.25 (*p* = 0.0089), successfully meeting the predefined noninferiority margin. In terms of safety, the incidence of grades 3 and 4 AEs was significantly lower in the THP group than in the TCbHP group (20.7% vs. 34.6%). Hematologic toxicities, such as neutropenia (6.8% vs. 16.4%) and leukopenia (5.5% vs. 14.8%), were markedly reduced. These results confirm that the THP regimen is noninferior to the TCbHP regimen in terms of pCR benefit.

In addition to chemotherapy de‐escalation, ADCs have further revolutionized the neoadjuvant landscape. In 2025, several studies, led by DESTINY‐Breast 11, signaled a full‐scale move toward the “ADC‐dominated era” in neoadjuvant treatment for HER2‐positive BC. The latest data from the DESTINY‐Breast 11 study which included 927 untreated patients with high‐risk HER2‐positive EBC (defined as T3–T4, N1–N3, or inflammatory BC) was reported at the 2025 ESMO Congress. The study compared eight cycles of T‐DXd, four cycles of T‐DXd followed by four cycles of THP (T‐DXd‐THP), and the standard four cycles of dose‐dense AC followed by four cycles of THP (ddAC‐THP). Results showed that the pCR rate in the T‐DXd‐THP group reached 67.3%, compared with 56.3% in the ddAC‐THP group. The difference in pCR rates was 11.2% (95% CI, 4.0–18.3; *p* = 0.003), indicating a statistically significant and clinically meaningful improvement. Furthermore, the overall safety profile of the T‐DXd‐THP group was superior, with ≥ grade 3 AEs occurring in 37.5% of patients compared with 55.8% in the ddAC‐THP group. These findings highlight the potential for ADCs to replace traditional, intensive anthracycline‐based chemotherapy regimens [31].

### 3.2 Progress in Adjuvant Therapy for HER2‐Positive BC

For patients who do not achieve a pCR (non‐pCR) following NAT, postoperative intensive adjuvant treatment is paramount for preventing distant metastasis. The KATHERINE study established trastuzumab emtansine (T‐DM1) as the standard regimen for this population [65]. However, the interim results of the DESTINY‐Breast 05 study, presented at the 2025 ESMO Congress, have further revolutionized the adjuvant treatment standards for HER2‐positive BC [32]. As the first head‐to‐head study comparing two ADCs in early‐stage BC, DESTINY‐Breast 05 aimed to evaluate the efficacy and safety of T‐DXd versus T‐DM1 in high‐risk HER2‐positive EBC patients with residual invasive disease after NAT. The results demonstrated that T‐DXd significantly reduced the risk of invasive disease recurrence or death by 53% compared with T‐DM1. The 3‐year iDFS rate was markedly higher in the T‐DXd group at 92.4%, compared with 83.7% in the T‐DM1 group. In terms of safety, the incidence of interstitial lung disease (ILD) was generally manageable and predominantly low‐grade. This study further validates the superior clinical benefit of T‐DXd in the intensified adjuvant offsetting for HER2‐positive EBC, indicating that T‐DXd is likely to reshape the postoperative standard of care for high‐risk early‐stage disease.

### 3.3 Progress in Treatment for HER2‐Positive ABC

#### 3.3.1 First‐Line Treatment for Advanced HER2‐Positive BC

The first‐line therapeutic landscape for HER2‐positive ABC is undergoing a profound paradigm shift, transitioning from the long‐standing dominance of standard THP regimens toward more potent, molecularly driven combinations. This evolution is characterized by three distinct clinical strategies: TKI intensification, ADC frontline substitution, and triplet maintenance escalation. The phase III PHILA trial has revolutionized the first‐line treatment landscape by demonstrating the superiority of the pyrotinib, trastuzumab, and docetaxel (PyroHT) regimen over the standard HT group [66]. Long‐term survival results from the PHILA study were further updated at the 2025 San Antonio Breast Cancer Symposium [33]. As of May 30, 2025, with a median follow‐up of 46.5 months in the PyroHT group and 44.6 months in the HT group, the median OS had not yet been reached in either arm. However, the PyroHT group demonstrated a significant OS benefit (HR = 0.74; 95% CI, 0.56–0.98; *p* = 0.0188). Notably, the incidence of all AEs decreased significantly following the discontinuation of docetaxel. These updated findings confirm that the pyrotinib‐based dual‐target regimen provides sustained OS and PFS benefits, further supporting its role as a key first‐line strategy.

The therapeutic paradigm for HER2‐positive ABC is being further revolutionized by the DESTINY‐Breast 09 study, which challenged the decade‐long dominance of the THP regimen [34]. As of February 26, 2025, the median PFS was 40.7 months in the T‐DXd plus pertuzumab (T‐DXd + P) group versus 26.9 months in the THP group (HR = 0.56; 95% CI, 0.44–0.71; *p* < 0.00001). The ORR was 85.1% for the T‐DXd + P arm compared with 78.6% in the THP group, with a median duration of response of 39.2 and 26.4 months, respectively. The safety profile was consistent with previous reports. In the T‐DXd + P arm, drug‐related ILD or pneumonitis occurred in 12.1% of patients (mostly grades 1 and 2). Key subgroup analyses presented at the 2025 ESMO Congress indicated that HR status and *PIK3CA* mutation status did not impact the efficacy of the T‐DXd + P regimen [35]. Furthermore, Asian subgroup data presented at the 2025 ESMO Asia Congress showed a median PFS of 40.7 months versus 27.4 months, reflecting a 45% reduction in the risk of progression or death (HR = 0.55), consistent with global population [36].

Currently, standard first‐line maintenance therapy consists of targeted agents following induction chemotherapy. Despite its efficacy, most patients ultimately experience disease progression. The HER2CLIMB‐05 study evaluated the addition of tucatinib to HP maintenance therapy after 4–8 cycles of taxane‐based induction. A total of 654 patients were randomized to receive either tucatinib or placebo combined with HP [37]. As of September 5, 2025, the tucatinib combination significantly improved PFS compared with placebo (HR = 0.641; 95% CI, 0.514–0.799; *p* < 0.0001), with consistent benefit observed across all subgroups. OS data are not yet mature. The most common treatment‐emergent AEs in the tucatinib group included diarrhea (72.7%) and elevated liver enzymes. Overall, the HER2CLIMB‐05 trial demonstrated that incorporation of tucatinib into first‐line maintenance therapy significantly improves PFS for patients with HER2‐positive ABC.

#### 3.3.2 Second‐Line and Subsequent Treatment for Advanced HER2‐Positive BC

In the second‐line and subsequent management of HER2‐positive ABC, next‐generation domestic ADCs are rapidly rewriting clinical standards by presenting formidable efficacy over historical controls. Two landmark phase III trials presented at the 2025 ESMO Congress highlight this therapeutic advance. First, the HORIZON‐Breast01 trial evaluated trastuzumab rezetecan (SHR‐A1811) versus the standard pyrotinib plus capecitabine doublet in patients previously treated with anti‐HER2 therapies [38]. Under rigid BICR assessment, SHR‐A1811 achieved an unprecedented therapeutic breakthrough, nearly quadrupling the median PFS to 30.6 months compared with 8.3 months in the control arm (HR = 0.22; *p* < 0.0001), alongside a substantial ORR of 81.7% and a highly favorable dose‐maintenance profile. Although the median duration of treatment for SHR‐A1811 was twice as long as the control (19.2 months), the incidence of TRAEs leading to dose reduction or interruption was notably lower. Second, the phase III trial of trastuzumab botidotin (A166) head‐to‐head challenged the historical standard T‐DM1 in taxane‐ and trastuzumab‐pretreated ABC. A166 significantly reduced the risk of disease progression or death by 61%, extending median PFS from 4.4 to 11.1 months (HR = 0.39; *p* < 0.0001) and markedly improving ORR (76.9% vs. 53.0%). A166 exhibited a differentiated safety profile; while requiring standardized ophthalmic monitoring for predictable corneal events, it demonstrated lower hematologic, hepatic, and gastrointestinal toxicities than T‐DM1 [39]. These phase III trials highlight the expanding role of next‐generation ADCs in previously treated HER2‐positive ABC, demonstrating substantial efficacy together with distinct and generally manageable safety profiles.

## 4 TNBC

TNBC is the most aggressive subtype of BC, accounting for nearly 15% of all BC cases worldwide. According to the results of the KEYNOTE‐522 trial, adding pembrolizumab to neoadjuvant chemotherapy improved the pCR rate, event‐free survival (EFS), and OS in patients with high‐risk early‐stage TNBC [67, 68]. However, in many resource‐limited regions, completing a full course of immunotherapy remains a significant challenge. Despite these advances, substantial unmet clinical needs remain, particularly for patients with advanced TNBC. Despite these advances, substantial unmet clinical needs remain, particularly for patients with advanced TNBC. Encouragingly, the 2025 results from the ASCENT‐03 and ASCENT‐04 trials demonstrated that the TROP2 ADC SG achieved favorable efficacy, safety, and QoL as a first‐line treatment for TNBC. These findings support the expanding role of TROP2‐directed ADCs in the first‐line treatment of advanced TNBC.

### 4.1 Progress in NAT for TNBC

For high‐risk early‐stage TNBC, combined or sequential neoadjuvant chemotherapy based on anthracyclines and taxanes has long been the standard of care. In recent years, several randomized controlled trials have demonstrated that the addition of platinum agents to neoadjuvant regimens can significantly improve pCR rates. The phase II HELEN‐001 aimed to evaluate the efficacy and safety of docetaxel plus cisplatin (TP) versus docetaxel, adriamycin, and cyclophosphamide (TAC) in patients with early‐stage TNBC [40]. The study enrolled 212 Asian female patients, who were randomized to receive either the TP or TAC regimen. Results showed that the pCR rate in the TP group was higher than in the TAC group (51.9% vs. 35.8%; *p* = 0.028). After a median follow‐up of 40 months, the 4‐year EFS rates were 86.1% in the TP group and 80.0% in the TAC group (HR = 0.639; *p* = 0.196). Notably, among patients harboring g*BRCA1/2* mutations, the TP group demonstrated significantly superior EFS compared with the TAC group (100% vs. 53.8%; *p* = 0.008). AEs were generally manageable in both groups, with ≥ grade 3 AE rates of 54% and 48% in the TP and TAC groups, respectively. This study indicates that the TP regimen achieves a higher pCR rate with manageable tolerability in the neoadjuvant setting.

Neoadjuvant immunotherapy has revolutionized the management of TNBC, yet dose optimization remains a critical clinical inquiry. The phase II PLANeT study challenged the conventional standard‐dose paradigm by evaluating low‐dose pembrolizumab (50 mg q6w) in combination with dose‐dense chemotherapy [41]. The low‐dose regimen achieved a pCR rate of 53.8%, numerically higher to the 40.5% observed in the control group, while maintaining a favorable safety profile with a lower incidence of grade ≥ 3 AEs (50.0% vs. 59.5%). These results indicate that dose‐reduced pembrolizumab warrants further investigation as a potentially more accessible neoadjuvant strategy, offering a promising, cost‐effective strategy to improve clinical accessibility and long‐term tolerability in TNBC management.

### 4.2 Progress in Adjuvant Therapy for TNBC

The optimization of adjuvant chemotherapy for TNBC remains heavily centered on re‐evaluating the therapeutic value of platinum intensification, which currently presents a nuanced landscape with conflicting clinical evidence. The phase III NRG‐BR003 trial challenged this approach by demonstrating that adding carboplatin to a dose‐dense anthracycline‐taxane backbone failed to improve 5‐year iDFS in node‐positive or high‐risk node‐negative TNBC (82.7% vs. 77.8%; HR = 0.78; *p* = 0.12), while significantly compounding systemic toxicity [42]. However, two major Asian phase III trials provided compelling, counterbalancing evidence. The multicenter RJBC‐1501 study conducted in China confirmed that incorporating carboplatin into an anthracycline‐taxane regimen significantly reduced the risk of recurrence by 34% (HR = 0.66; *p* = 0.034) and the risk of death by 61% (HR = 0.39; *p* = 0.029), exhibiting a manageable safety profile despite expected hematologic toxicities [43]. Similarly, the CITRINE study, which restricted eligibility to high‐risk early TNBC (node‐positive or Ki‐67 ≥ 50%), demonstrated that a carboplatin‐intensified triplet significantly improved both 3‐year DFS (92.3% vs. 85.8%; HR = 0.64) and 3‐year OS (98.0% vs. 94.0%; HR = 0.41) [44]. Notably, the survival curves in CITRINE diverged primarily within the first year post‐treatment, suggesting that platinum agents improve long‐term prognosis primarily by suppressing the characteristically high risk of early recurrence in biologically aggressive disease. These contrasting data sets underscore that the utility of adjuvant platinum escalation is highly dependent on precise patient selection, with high‐risk, highly proliferative, and Asian cohorts deriving the most definitive survival advantages.

### 4.3 Progress in Treatment for Metastatic TNBC

The first‐line therapeutic paradigm for metastatic TNBC (mTNBC) is undergoing a major clinical evolution, driven by the programmatic migration of TROP2‐directed ADCs into earlier lines of therapy. This shift is executed through two distinct approaches: establishing ADC monotherapy as a front‐line chemotherapy replacement for immunotherapy‐ineligible cohorts, and leveraging ADC‐immunotherapy synergy to enhance durable remission in PD‐L1‐positive disease.

First, in populations unsuitable for immunotherapy, the phase III ASCENT‐03 and TROPION‐Breast02 trials have successfully mounted a dual challenge to historical standards. On the basis of the results of the ASCENT trial, SG has been approved in multiple countries for mTNBC patients who have received at least two prior systemic therapies (or at least one for metastatic disease) [69]. The ASCENT‐03 study further focused on patients with PD‐L1‐negative mTNBC or those ineligible for immunotherapy, providing robust evidence for SG monotherapy in the first‐line setting [45]. The study enrolled 588 patients and showed that the mPFS was 9.7 months in the SG group versus 6.9 months in the chemotherapy group, with a stratified hazard ratio of 0.62 (*p* < 0.001). Regarding survival data, the median OS was 21.5 months in the SG group versus 20.2 months in the chemotherapy group, though OS data are not yet mature. Safety analyses indicated comparable AE rates, with any‐grade AEs occurring in 273 patients (99%) in the SG group and 269 patients (97%) in the chemotherapy group. Grade ≥ 3 AEs occurred in 181 (66%) and 171 (62%) patients, respectively. The TROPION‐Breast02 study, reported at the 2025 ESMO Congress, further confirmed datopotamab deruxtecan (Dato‐DXd) as a novel first‐line treatment option for patients with advanced TNBC who are ineligible for immunotherapy [46]. The results demonstrated that in this specific population, first‐line Dato‐DXd provided a significant PFS benefit over chemotherapy (10.8 months vs. 5.6 months; HR = 0.57; *p* < 0.0001), extending the median PFS by 5.2 months and reducing the risk of disease progression or death by 43%. Besides, Chinese‐developed TROP2 ADCs have also demonstrated promising efficacy. Following the OptiTROP‐Breast 01 study, which confirmed its efficacy in later‐line settings [70], the 2025 ASCO Annual Meeting presented results from the phase II OptiTROP‐Breast05 study [47]. This trial evaluated Sacituzumab Tirumotecan as a first‐line treatment for advanced TNBC, yielding an impressive median PFS of 13.4 months. In terms of safety, the AE profile and tolerability were consistent with previous reports. The most common grade ≥ 3 TRAEs included decreased neutrophil count (46.3%), decreased white blood cell count (34.1%), and anemia (12.2%), all of which were clinically manageable.

Second, for patients with PD‐L1‐positive or unselected biomarker status, the combination of TROP2 ADCs with immune checkpoint inhibitors represents the premier frontline strategy. The phase III ASCENT‐04 trial demonstrated that combining SG with pembrolizumab significantly prolonged median PFS over standard chemotherapy‐pembrolizumab triplets (11.2 vs. 7.8 months; HR = 0.65; *p* = 0.0009) [48]. The safety findings were consistent with the known toxicities of each agent. Overall, the SG plus pembrolizumab regimen was better tolerated, with lower rates of treatment‐emergent AEs leading to treatment discontinuation (12% vs. 31%) or dose reduction (35% vs. 44%). Similarly, the updated phase II BEGONIA trial showed that the Dato‐DXd plus durvalumab achieved a profound ORR of 79.0% and extended the median PFS to 14.0 months. In the PD‐L1 high‐expression subgroup, the combination showed an even higher ORR of 81.8%. The safety profile remained manageable, with a low incidence of ILD (3%–5%) and all of which was grade 1 or 2 [49]. These landmark advances confirm a comprehensive paradigm shift toward ADC‐centered frontline management, effectively surpassing the therapeutic limitations of traditional chemotherapy in metastatic luminal‐negative disease.

## 5 Exploration of Artificial Intelligence (AI) Applications in BC

With the continued global increase in cancer incidence, the long‐term management of malignant tumors has become an urgent priority. In BC, loss to follow‐up in early‐stage patients and insufficient adherence monitoring in advanced‐stage patients often result in poorer prognoses. The 2025 ESMO AI Congress reported an artificial intelligence‐driven follow‐up platform for BC [71]. The platform integrates the ChatGLM model, national clinical guidelines, patient education materials, and real‐world data. By utilizing virtual avatars and voice‐cloning technology to drive physician‐side mini‐programs, the system facilitates expert‐level patient interaction and automates follow‐up management. The application was pioneered in two distinct clinical scenarios: follow‐up adherence and QoL assessment for early‐stage BC patients, and treatment adherence and AE monitoring for advanced‐stage patients receiving ADCs. The platform's functionalities encompass emotional support, risk assessment, reminders, report interpretation, rehabilitation planning, follow‐up scheduling, and medical record summarization. The reported results indicated that AI‐based medication guidance reduced initial consultation time by 60%, while automated reporting led to a 50% reduction in outpatient volume. Furthermore, the digital rehabilitation module increased patient education coverage by 45%, and proactive AE monitoring improved patient satisfaction by 35%. These findings suggest that implementation of this AI‐enhanced follow‐up platform can significantly improve patient satisfaction and reduce unplanned clinic visits, providing a scalable and transferable framework for AI‐assisted oncology care across diverse global healthcare systems.

## 6 Conclusions

The past few years have witnessed a systemic shift in BC treatment paradigms, characterized by continuous innovation. Combination strategies involving ET, targeted therapy, immunotherapy, and chemotherapy are undergoing continuous optimization, providing evidence‐based support for the clinical management of different molecular subtypes. In HR‐positive/HER2‐negative BC, innovative agents continue to move into earlier lines of therapy, significantly expanding the therapeutic armamentarium. The treatment of HER2‐positive BC is progressively transitioning toward an ADC‐driven therapeutic paradigm. Meanwhile, in TNBC, synergistic strategies combining ADCs with immunotherapy are carving out entirely new avenues for improving patient survival. These advances are driving BC treatment toward a more precise, efficient, and patient‐centered model of long‐term management.

## Author Contributions

**Xuenan Peng:** writing – original draft, writing – review and editing. **Yujing Tan:** writing – original draft, writing – review and editing. **Fei Ma:** conceptualization, supervision.

## Ethics Statement

The authors have nothing to report.

## Consent

The authors have nothing to report.

## Conflicts of Interest

Professor Fei Ma is a member of the *Cancer Innovation* Editorial Board. To minimize bias, he was excluded from all editorial decision‐making related to the acceptance of this article for publication. The remaining authors declare no conflicts of interest.

## Data Availability

The data for this work may be shared upon reasonable request to the corresponding author.
